# Synthesis and characterization of noble metal–titania core–shell nanostructures with tunable shell thickness

**DOI:** 10.3762/bjnano.8.208

**Published:** 2017-10-05

**Authors:** Bartosz Bartosewicz, Marta Michalska-Domańska, Malwina Liszewska, Dariusz Zasada, Bartłomiej J Jankiewicz

**Affiliations:** 1 Institute of Optoelectronics, Military University of Technology, Kaliskiego 2 Str. 00-908 Warsaw, Poland; 2 Faculty of Advanced Technologies and Chemistry, Military University of Technology, Kaliskiego 2 Str. 00-908 Warsaw, Poland

**Keywords:** Ag@TiO_2_, Au@TiO_2_, core–shell nanostructures, titania coating, titanium dioxide, tunable resistive pulse sensing

## Abstract

Core–shell nanostructures have found applications in many fields, including surface enhanced spectroscopy, catalysis and solar cells. Titania-coated noble metal nanoparticles, which combine the surface plasmon resonance properties of the core and the photoactivity of the shell, have great potential for these applications. However, the controllable synthesis of such nanostructures remains a challenge due to the high reactivity of titania precursors. Hence, a simple titania coating method that would allow better control over the shell formation is desired. A sol–gel based titania coating method, which allows control over the shell thickness, was developed and applied to the synthesis of Ag@TiO_2_ and Au@TiO_2_ with various shell thicknesses. The morphology of the synthesized structures was investigated using scanning electron microscopy (SEM). Their sizes and shell thicknesses were determined using tunable resistive pulse sensing (TRPS) technique. The optical properties of the synthesized structures were characterized using UV–vis spectroscopy. Ag@TiO_2_ and Au@TiO_2_ structures with shell thickness in the range of ≈40–70 nm and 90 nm, for the Ag and Au nanostructures respectively, were prepared using a method we developed and adapted, consisting of a change in the titania precursor concentration. The synthesized nanostructures exhibited significant absorption in the UV–vis range. The TRPS technique was shown to be a very useful tool for the characterization of metal–metal oxide core–shell nanostructures.

## Introduction

In recent years, core–shell nanostructures (CSNs) have become one of the most widely studied hybrid structures [1–2]. This is because the combination of two or more different materials into one structure of controlled size, geometry and morphology can lead to either improved or new properties not observed in the individual constituent materials. CSNs with a silica core and noble metal shell exhibiting tunable optical properties depending on the ratio of core radius and shell thickness are an excellent example of such structures [3–4]. The CSNs, with either a silica core or shell, have found many applications due to their useful properties, including surface enhanced spectroscopy or cancer therapy [4–11]. For many applications, however, the use of titanium dioxide in CSNs would be of much greater interest. Useful physicochemical properties of titanium dioxide in its crystalline forms, rutile and anatase, such as high refractive index and photocatalytic activity have led to its use in many fields. For example, TiO_2_ nanomaterials have been investigated for their use in photocatalysis [12–14], photocatalytic fuel generation [15], photovoltaics and sensors [16–17]. The CSNs with noble metal (Au, Ag) nanoparticles (NPs) as a core and TiO_2_ shell, Au@TiO_2_ and Ag@TiO_2_, have great potential for use in these applications [18–19]. Surface plasmon resonance properties of gold and silver NPs can increase the optical absorption of titania and extend its absorption band to the visible light region. Such CSNs could allow one of the most important limitations in broader use of titania to be overcome: the limitation of photocatalytic capability to UV light (λ < 400 nm). In addition, they may serve as a precursor for plasmon-sensitized colloidal perovskites, which are materials of great interest for solar cell applications [20].

The limiting factor in the broader use of Ag@TiO_2_ and Au@TiO_2_ structures could be their rather difficult synthesis process [21–22]. The main problem in coating various particles (including metal colloids) with titania is the very fast hydrolysis rate of its most commonly used precursors, titanium alkoxides, which makes the coating process hard to control [21]. This is also the main reason for the low monodispersity of TiO_2_ particles prepared from titanium alkoxides using the sol–gel method [23]. The limitations in titania coating can be overcome by using the less common but more expensive TiO_2_ precursors, various solvents or their mixtures, various additives such as surfactants or salts, and special reaction conditions in order to slow down the reaction rate. These strategies have been employed in a few successful attempts at controlled synthesis of Ag@TiO_2_ and Au@TiO_2_ structures reported in recent years [21,24–49]. However, these titania coating methods were used in the synthesis of either Ag@TiO_2_ [24–36] or Au@TiO_2_ [37–49] nanostructures with only a few exceptions of more general methods [21]. Another difficulty in the coating process arises from the possibility of core particle agglomeration. Since metal nanoparticles are vulnerable to agglomeration, additional actions, such as the use of special conditions or additives, have to be undertaken to prevent it. In addition, the confirmation of the shell formation (in almost all reported cases) has been based only on electron microscopy (mainly TEM) images and UV–vis spectroscopy, with a few exceptions of the additional use of either static or dynamic light scattering (SLS or DLS) [21,27].

**Scheme 1:**
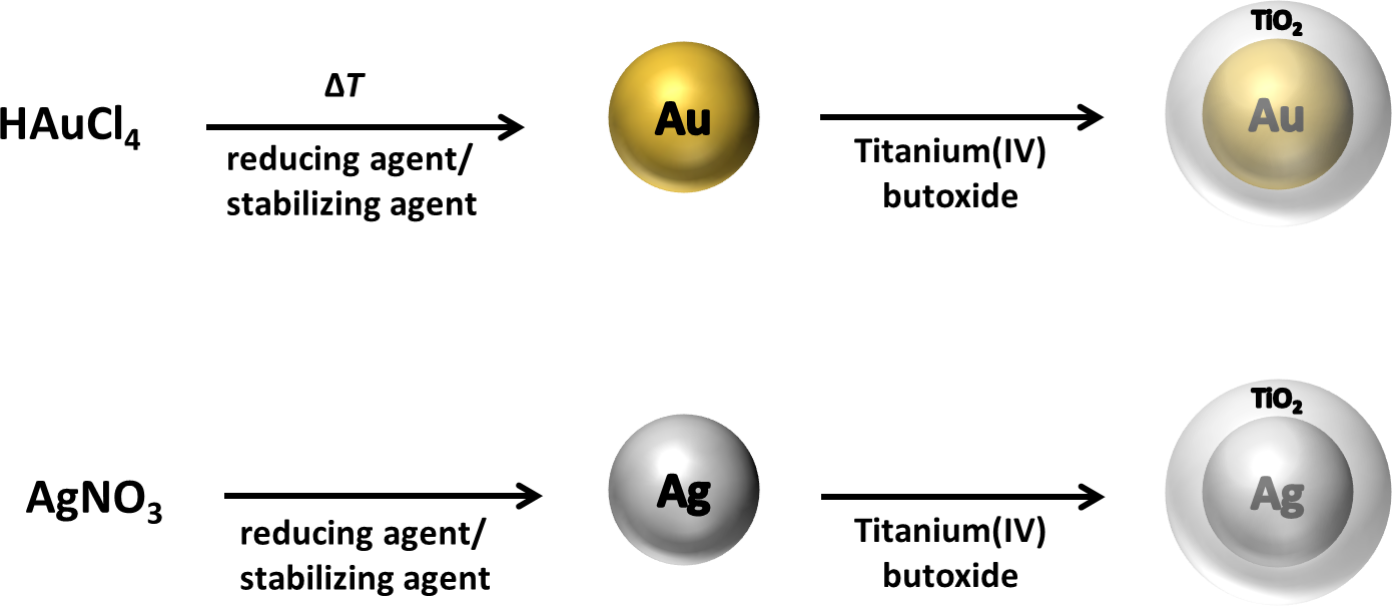
Synthesis route of the Au@TiO_2_ and Ag@TiO_2_ core–shell nanostructures.

Here, we report a general and simple approach to the synthesis of Ag@TiO_2_ and Au@TiO_2_ CSNs (Scheme 1). The proposed method works well for both gold and silver NPs without any additional adjustments and without the need for special reaction conditions. It also allows control of the shell thickness in the range of 20–30 nm up to 100 nm simply by changing the titania precursor concentration. These as-prepared materials have significant absorption in the UV and visible range and therefore have high potential for applications in solar-light-driven photocatalysis and photovoltaics. In addition, we show for the first time the potential of the tunable resistive pulse sensing (TRPS) technique in the characterization of metal-oxide CSNs. TRPS, which in a relatively easy and fast way provides statistical information regarding the size and size distribution of the studied particles, can be a valuable tool in the characterization of various nanoparticles in addition to electron microscopy.

## Results and Discussion

### Synthesis of Ag@TiO_2_ and Au@TiO_2_

**Figure 1:**
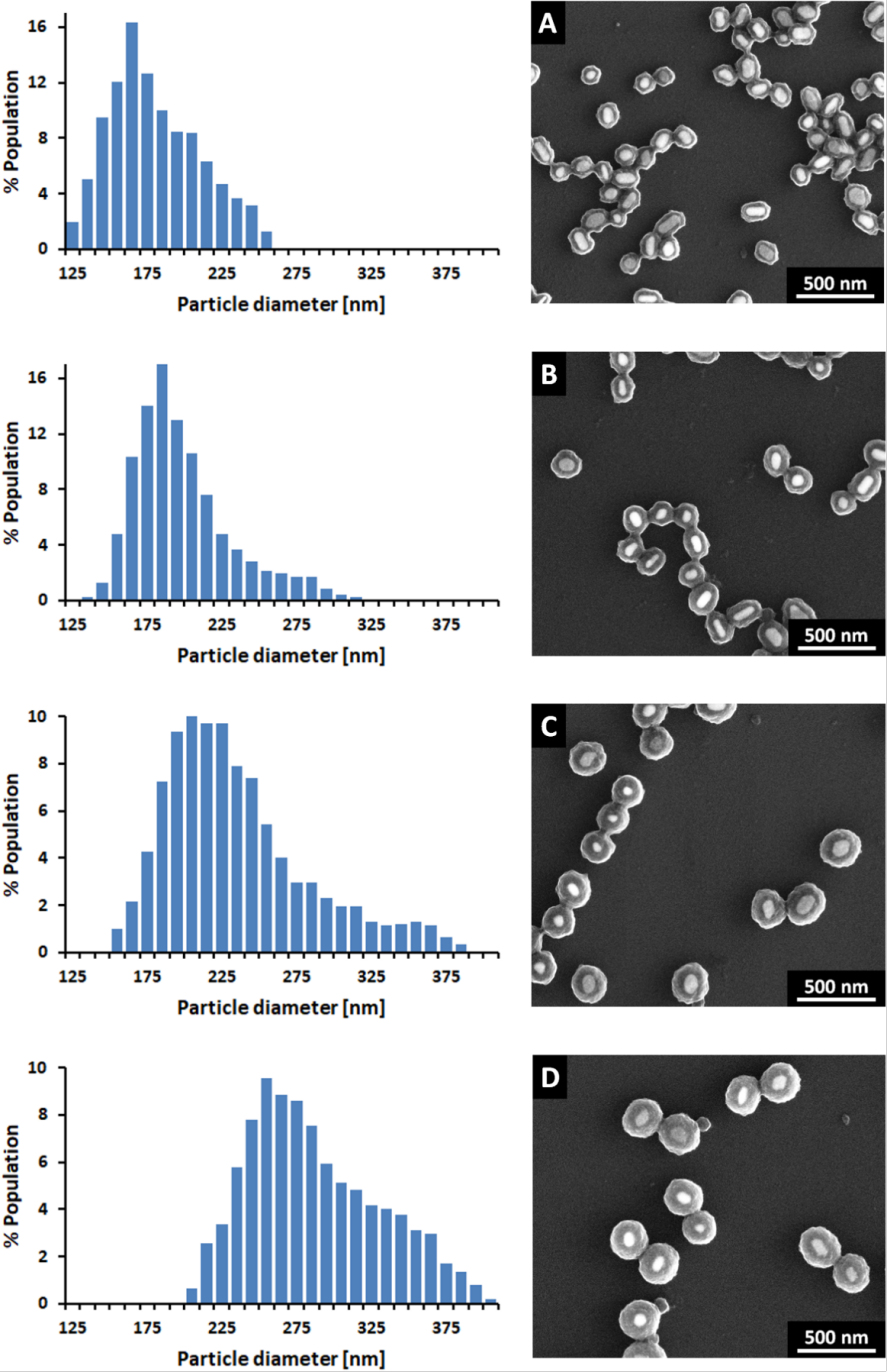
SEM images and TRPS size histograms of Au@TiO_2_ (Samples A–D) structures with various titania shell thicknesses (see Table 1).

Our studies on the fabrication of CSNs with a noble metal core and titania shell were aimed at the development of a general and simple method which requires a minimal number of additives (or none at all) and allows control of the structural features of the CSNs in addition to synthesis in larger amounts. The TiO_2_ coating strategy used in the synthesis of Ag@TiO_2_ and Au@TiO_2_ CSNs is outlined in Scheme 1. In the first step, we synthesized AuNPs using the Frens method and AgNPs by the reduction of silver nitrate with hydroxylamine hydrochloride [50–51]. In both cases, relatively monodisperse spherical or quasi-spherical metal NPs with a mean particle diameter of around 100 nm were obtained (Table 1, Figure 1 and Figure S1, Supporting Information File 1). Initially, we also used the Frens method to synthesize AgNPs. As a result, however, we obtained significant amounts of rod-like and triangular particles in addition to spherical particles. The titania coating method described here allowed coating of all AgNPs regardless of shape, but such CSNs were not very useful for further analysis from a statistical point of view and therefore were not presented in this article. In addition, we found that the methodology described here works very well for nanoparticles with diameters greater than 50 nm. In cases of smaller nanoparticles, with diameters of 20 nm and less, we observed formation of the multi-core@shell particles, which are also very interesting due to the combined plasmonic effect of metallic cores.

In the case of AgNPs, the synthesized metal NPs were stabilized with citrate ions either during the synthesis or after, in order to prevent aggregation in the coating solution. Citrate stabilized AuNPs and AgNPs disperse well in the reaction mixture used, ethanol and acetonitrile, and do not undergo undesirable aggregation. In previously reported studies metal nanoparticles were usually synthesized and stabilized in a separate step from coating [27–33,35–49]. However, in some of the reported methods, both the synthesis of NPs and their coating occurred in one reaction batch [24–26,34]. The former approach allows synthesis and coating of particles of various shapes and sizes. In the latter approach, control over the shape and size of the synthesized particles is limited. The metal nanoparticles were stabilized before the coating step using various surfactants or stabilizing agents such as Lutensol ON50 [21], cetyltrimethylammonium bromide (CTAB) [27–30,40], anionic poly(sodium 4-styrenesulfonate) (PSS) [49], mercaptoundecanoic acid [38–39], 1-ethenylpyrrolidin-2-one (PVP) [33,41], or hydroxypropyl cellulose [45–47]. In addition, in some studies, an adhesion layer was formed on the metal NP surface in order to better control the TiO_2_ shell growth [48].

The coating step of the method described here, being a modification of the titania particle synthesis method described elsewhere [52–53], small volumes of the concentrated aqueous suspensions of synthesized NPs were transferred into a mixture of ethanol and acetonitrile. The hydrolysis reaction catalyst, methylamine, was then added to a suspension of NPs. The role of the amine catalysts is to transfer a proton from the water molecule to the oxygen atoms in titanium alkoxide molecules. As a result, good leaving groups of alcohols are formed, which are then easily replaced by hydroxide anions. In the next step, titanium(IV) butoxide (TBT) solution in absolute ethanol was added dropwise. The first indication of the start of the coating process was observed shortly after precursor addition; however, the reaction was allowed to proceed for a few hours to ensure formation of the complete shell. The shell formation on metal NPs is accompanied by the unavoidable formation of free TiO_2_ particles, which together with residual chemicals, are removed by a few centrifugation/wash/redispersion cycles in ethanol.

The method described here for titania coating of metal NPs is simple and does not require special conditions. The reaction is carried out at room temperature without the need for an inert atmosphere. However, due to the sensitivity of titanium alkoxides to water, care should still be taken while handling the TiO_2_ precursor. This is one of the reasons why we used TBT in our method as a shell precursor instead of titanium(IV) isopropoxide or titanium(IV) ethoxide. We found that TBT is more stable and reacts slower than titania precursors with smaller alkyl groups. This finding is in agreement with previously reported results, indicating that hydrolysis and condensation rates of titanium alkoxides decrease when the alkyl group size increases due to the partial charge and steric effects [54–55]. In previously reported studies on titania coating, in order to achieve better control of the coating process, titanium alkoxides were converted to titanium glycolate before coating [35,56] or coating was carried out using less common TiO_2_ precursors such as, titanium(IV) bis(ammonium lactato) dihydroxide [37] or titanium diisopropoxide bis(acetylacetonate) [45–47]. TBT also has an additional advantage: it is cheaper than other TiO_2_ precursors which could be an important factor when considering scaling up of the synthesis. Despite the relative stability of TBT, the use of anhydrous solvents was necessary in order to avoid premature hydrolysis of TBT in the ethanol solution, and also to avoid the uncontrolled introduction of water to the reaction mixture. Water, necessary for titania precursor hydrolysis, is introduced to the reaction mixture in a controlled way from two sources, NPs suspensions (≈199 µL) and amine catalysts solutions (≈24 µL of water), before addition of the shell precursor. The total volume of added water to the reaction mixture was kept constant, which allowed the control of thicknesses of the titania coating by only varying the TBT concentration in the final reaction mixture (Table S1, Supporting Information File 1).

### Morphology, size and shell thickness of Ag@TiO_2_ and Au@TiO_2_

The characterization of the structural features, size and shell thickness of CSNs, such as those described in this article, is complicated. Traditionally, only electron microscopy techniques (TEM or SEM) and UV–vis spectroscopy have been used to prove that the coating was in fact achieved and to provide information regarding shell thickness [21,24–49,57]. A few exceptions concerning the additional use of SLS or DLS have also been reported [21,27]. However, any statistical data on the CSN size and shell thickness have been based only on the analysis of transmission electron microscopy images due to the high resolution of this technique. Recently, it has been demonstrated that of the various particle sizing techniques, two of them (differential centrifugal sedimentation (DCS) and tunable resistive pulse sensing (TRPS) [58–59]) are capable of achieving resolution similar to the TEM technique. Both techniques allow the analysis of many more particles than TEM, and thus better statistics are obtained in a simpler way, in shorter time and much more cheaply. The main drawback of these techniques is that they cannot provide information regarding the shape of particles and should therefore always be used together with either TEM or SEM. The DCS technique has already been employed to investigate some types of CSNs, but these studies were possible due to knowledge of the density values of both core and shell, which are necessary for the analysis [60–62]. For the same reason, the use of the DCS technique for the characterization of fabricated CSNs was not possible in our case due to the lack of the shell density value, necessary for the determination of particle diameter based on Stokes’ Law. Regarding the CSNs studied, we found that the TRPS technique could be a very valuable addition to electron microscopy techniques in the characterization of CSNs. TRPS, based on the Coulter principle, monitors changes in ionic current as individual particles pass through an elastomeric membrane containing a single pore of precisely controlled size. Because TRPS allows measurement particle-by-particle, any central value or spread statistic based on hundreds or thousands of individual measurements can be calculated and transformed for direct comparison with ensemble average data. In contrast to electron microscopy, TRPS does not entail difficult sample preparation and experimental artefacts, and is significantly cheaper. More importantly, TRPS measurements are independent of particle density and optical properties such as particle labelling or refractive index [63–65]. In fact, it is the only technique among more common and cheaper ones which allows such valuable statistical data to be obtained in the case of CSNs.

**Figure 2:**
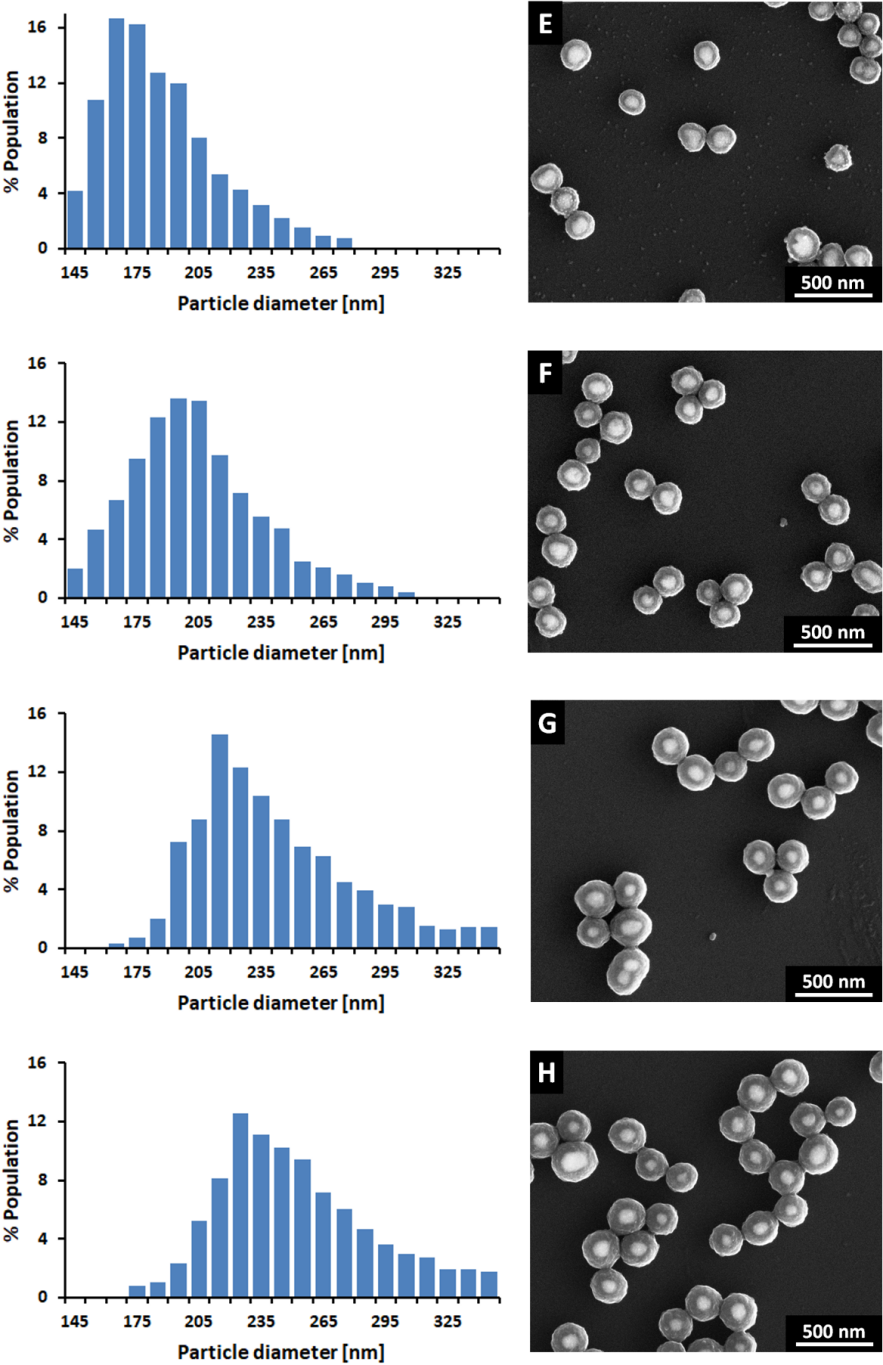
SEM images and TRPS size histograms of Ag@TiO_2_ (Samples E–H) structures with various titania shell thicknesses (see Table 1).

SEM images and TRPS size histograms of synthesized Au@TiO_2_ and Ag@TiO_2_ CSNs are presented in Figure 1 and Figure 2, respectively. TRPS size histograms of NPs are presented in Figure S1, Supporting Information File 1. The detailed statistical data regarding size and shell thicknesses of synthesized nanostructures, mean (*D*_P_ mean), mode (*D*_P_ mode), median (*D*_P_50), maximum (*D*_P_ max) and minimum (*D*_P_ min) values of core particles and CSNs diameters as well as the shell thicknesses (*d*) calculated based on these values are provided in Table 1. Authors very often do not provide sufficient information regarding size distribution, giving only mean size with standard deviation. This suggests that size distribution means Gaussian distribution, which in many cases does not reflect real size distribution. Therefore, the inclusion of other size parameters in order to describe the width of the distribution such as values of mean, median and mode diameters is recommended. The mean diameter is a calculated value similar to the concept of average and provides information regarding the average size of all measured particles. Median diameter value is defined as the value where half of the particle population has a smaller size, and half has a larger size. The mode value represents the particle size (or size range) most commonly found in the distribution. For symmetric distributions such as the one shown in Supporting Information File 1, Figure S1 and Table 1 for AuNPs, all values, mean, median and mode, are equivalent. However, the situation changes upon titania coating. In the case of both Au@TiO_2_ and Ag@TiO_2_ CSNs, TRPS size histograms indicate that with increasing shell thickness the non-uniformity of the size distribution with respect to metallic cores (Figure 1, Figure 2 and Supporting Information File 1, Figure S1) also increases. This finding was not easily observed based on the analysis of SEM images with a small number of particles. In order to obtain good statistical data, a SEM analysis would require a time-consuming investigation of the many SEM images with a larger number of CSNs.

**Table 1:** Particle size and particle size distribution of synthesized core–shell nanostructures measured using TRPS.^a^

Samples	*D*_P_ mean [nm]	*D*_P_ mode [nm]	*D*_P_50 [nm]	*D*_P_ min [nm]	*D*_P_ max [nm]	*d* mean [nm]^b^	*d* mode [nm]^c^	*d D*_P_50 [nm]^d^	*d* min [nm]^e^	*d* max [nm]^f^

Au NPs	101	98	99	77	143	–	–	–	–	–
A	192	165	189	121	253	45	33	45	22	55
B	205	185	193	126	313	52	43	47	25	85
C	244	195	227	152	384	71	48	64	38	121
D	300	245	284	202	401	99	73	93	63	129
Ag NPs	116	111	113	82	160	–	–	–	–	–
E	188	165	180	141	273	36	27	34	30	57
F	204	204	200	142	303	44	46	44	30	72
G	243	215	234	162	341	63	52	61	40	91
H	261	225	249	170	343	72	57	68	44	92

^a^*D*_P_ – particle diameter; *d* – shell thickness. The shell thickness obtained based on the values of ^b^mean *D*_P_, ^c^mode *D*_P_, ^d^*D*_P_50 (median), ^e^minimum *D*_P_ (*D*_P_ min) and ^f^maximum *D*_P_ (*D*_P_ max).

The results obtained using SEM and TRPS, as shown in Figure 1, Figure 2, Table 1 and Supporting Information File 1, Table S1, clearly show that increasing the titania precursor concentration in the final reaction mixture results in thicker titania shells. In the case of AgNPs with *D*_P_50 = 113 nm under the applied reaction conditions (Supporting Information File 1, Table S1), the titania shell thickness changes from 34 nm to 68 nm based on the *D*_P_50 values of the diameters. A similar range of titania shell thickness values is obtained based on the *D*_P_ mean values of the diameters. In *D*_P_ mode values, all but one value of the shell thickness is smaller than the corresponding *d* values calculated for *D*_P_50 and *D*_P_ mean. In the case of AuNPs with *D*_P_50 = 99 nm under the applied reaction conditions (Supporting Information File 1, Table S1), the titania shell thickness changes from 45 nm to 93 nm based on the *D*_P_50 values of the diameters. A similar range of the titania shell thickness values is obtained based on the *D*_P_ mean values of the diameters. In the case of the *D*_P_ mode, all values of shell thickness are smaller than the corresponding values calculated for *D*_P_50 and *D*_P_ mean.

Under certain assumptions, analysis of the *d* values calculated based on the *D*_P_ max and *D*_P_ min (the largest and the smallest particles in the samples) can provide information about the theoretical range of the titania shell thickness values for each series of CSNs, Samples A–H. The analysis of the SEM images of the CSNs investigated revealed that for the AgNPs and AuNPs of similar sizes, the shell thicknesses seem to be very uniform. We have not observed CSNs with titania shell thicknesses which were much larger or smaller compared to shell thicknesses of other CSNs. We are aware, however, that a small number of CSNs in the population may have much thinner or much thicker shells. Based on these findings we have assumed in our calculations that metal NPs with *D*_P_ min yield CSNs with *D*_P_ min and metal NPs with *D*_P_ max yield CSNs with *D*_P_ max. Taking into consideration the above assumptions, the thinnest obtained titania shell was about 22 nm for AuNPs and about 30 nm for AgNPs. Interestingly, the value of *d* min increases more slowly for AgNPs than for AuNPs. A similar trend is observed for *d* max. The *d* max under the applied reaction conditions reaches almost 130 nm for AuNPs, while only 90 nm for AgNPs.

### Optical properties of Ag@TiO_2_ and Au@TiO_2_

**Figure 3:**
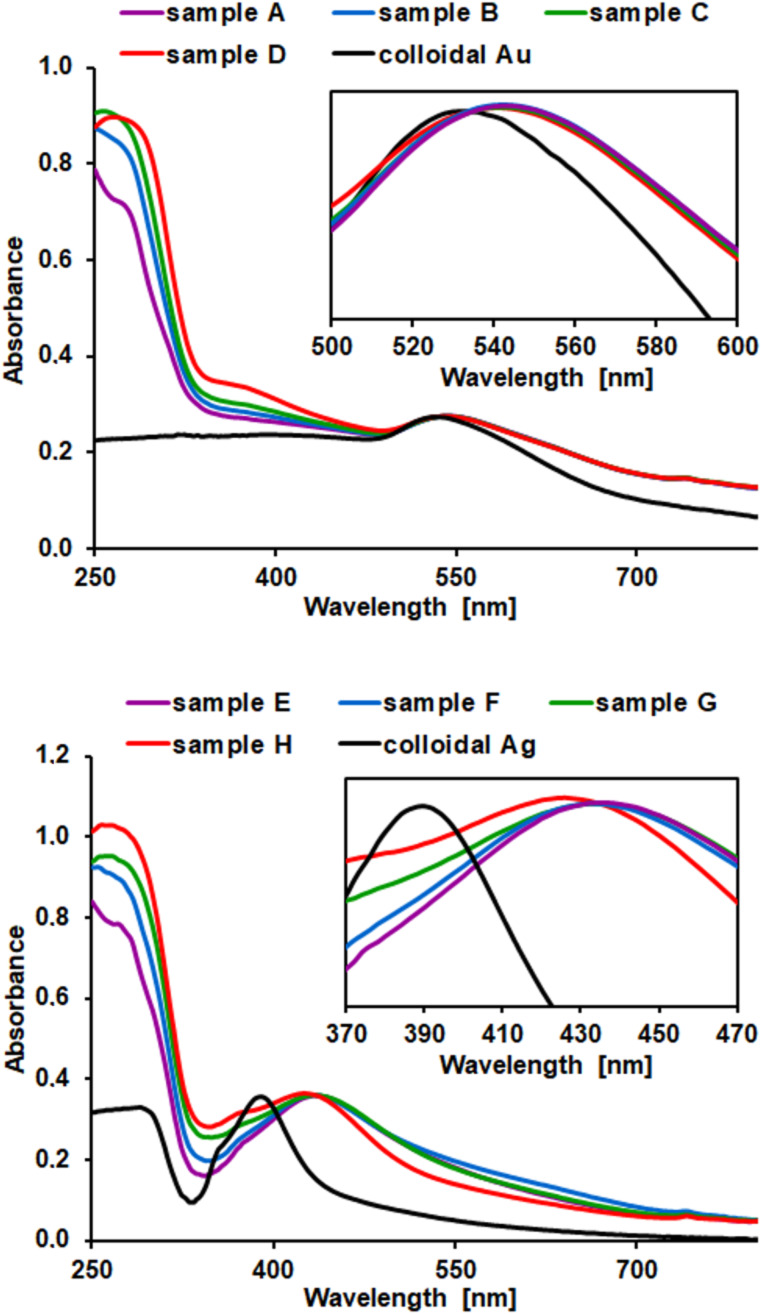
Normalized UV–vis spectra of noble metal colloids and noble metal@TiO_2_ core–shell nanostructures for Au (top) and Ag (down). All spectra were normalized to the peaks relative to the surface plasmon resonances of CSNs.

The UV–vis spectra of metal NPs and CSNs fabricated from them are shown in Figure 3. In order to better visualize the optical properties of the fabricated CSNs, images of their water suspensions and powders after drying are shown in Supporting Information File 1, Figure S2. In the case of the CSNs, we observed that upon coating of metal NPs with titania, the optical properties change significantly compared to the optical properties of the core and the shell material alone (Figure 3 and Supporting Information File 1, Figure S3).

The fabricated CSNs absorb light in the whole UV–vis range and therefore their optical properties compared to TiO_2_ particles are considered to be improved (Supporting Information File 1, Figure S3). In addition, when comparing the optical properties of metal NPs and the CSNs, red shifts of the maxima of absorption in CSNs (λ_max_ = 390 nm vs λ_max_ = 424 nm for AgNPs and λ_max_ = 528 nm vs λ_max_ = 540 nm for AuNPs) were observed (Figure 3). This effect is related to the fact that the spectral location of plasmon resonance of single noble metal nanoparticles is dependent on the refractive index (*n*) of the surrounding medium [28,56]. Coating metal NPs with TiO_2_ (*n* ≈ 2.2–2.6) leads to an overall increase in the refractive index of their local dielectric environment, and as a result, to the red shift of the plasmon resonance. In the case of both Ag@TiO_2_ and Au@TiO_2_, the changes in the shell thickness do not significantly influence the position of the plasmon resonance and overall extinction in the UV–vis range. However, an additional increase in the red shift of maxima of absorption is expected upon transformation of the amorphous titania shell to crystalline form.

Improved optical properties of titania-based hybrid nanostructures make them interesting materials for application in dye-sensitized solar cells (DSSCs) and photocatalysis. In fact, it has been shown that plasmonic nanostructures can enhance the efficiency of DSSCs by four possible mechanisms [66]. The far-field coupling of scattered light and the near-field coupling of electromagnetic fields increased the efficiency of light interaction with sensitizers (dyes). On the other hand, plasmon resonance energy transfer (PRET) and “hot” electron transfer led to an increased e^−^/h^+^ pair generation and amplified number of carriers available for photocurrent generation. An increased number of e^−^/h^+^ pairs should also result in improved photocatalytic properties of titania-based plasmonic nanostructures.

## Conclusion

In this paper, we have shown that by using a general and simple approach it is possible to synthesize Ag@TiO_2_ and Au@TiO_2_ CSNs with shell thickness of ≈40–70 nm and 90 nm, for AgNPs and AuNPs, respectively (based on the *D*_P_50 values). In the titania coating method developed, we used titanium(IV) butoxide, the least expensive of the organic titanium alkoxides, and used it under mild reaction conditions at room temperature and with no inert atmosphere or special glassware. The method was applicable to both gold and silver particles under exactly the same conditions and this allowed us to obtain relatively large amounts of the CSNs in a single batch. In previously reported studies, titania coating of noble metal nanoparticles was achieved by using various, more or less complicated approaches. These approaches included the use of the less common and thus more expensive titania precursors, which are laborious in preparation. The other approaches included the use of various solvents or their mixtures, various additives such as surfactants or salts, special reaction conditions, and multistep processes. In addition, these strategies were employed in the synthesis of either Ag@TiO_2_ or Au@TiO_2_ structures with some exceptions, including more general methods. Moreover, in all previous studies, information regarding the shell thickness was obtained mainly from the analysis of electron microscope images, which have some limitations in terms of the statistical analysis. In our studies, we applied the TRPS technique to characterize the metal–metal oxide core–shell nanostructures for the first time. This technique allowed us, in a very convenient and fast way, to analyze hundreds of CSNs per sample and to obtain very detailed statistical data regarding their size and size distribution. However, TRPS does not provide information about the shape of the investigated particles and in their analysis always has to be used as a complementary tool to electron microscopy techniques. Fabricated core–shell nanostructures have significant extinction in the UV and visible range and therefore should be of great interest for applications in solar-light-driven photocatalysis and photovoltaics. In the future, studies will be carried out to further optimize reaction conditions toward coating nanoparticles with diameter of less than 20 nm and to obtain thinner shells. In addition, studies will be dedicated to converting the titania shell of the synthesized CSNs to either crystalline titania (anatase, rutile or their mix) or perovskites and to testing the performance of such systems in various applications in comparison to regular titania or perovskite particles.

## Experimental

### Chemicals

All chemicals, including sodium citrate dihydrate (>99%), hydroxylamine hydrochloride (>99%), gold(III) chloride hydrate (99.99%) and titanium(IV) butoxide (>97%) were purchased from Sigma-Aldrich. Methylamine (40% w/w aq. soln.) and silver nitrate (99.9%) were purchased from Alfa Aesar. Ethanol (99.8%) and acetonitrile (99.5%) were purchased from Avantor Performance Materials Poland. Nitric acid (65% w/w aq. soln.), hydrofluoric acid (40% w/w aq. soln.) and sodium hydroxide (>99%) were purchased from Chempur. All purchased chemicals were used as received without further purification. Ultrapure deionized (DI) water (18.2 MΩ·cm at 25 °C, Hydrolab, Poland) was used throughout the experiments. All glassware was treated with titania etching solution (HF/HNO_3_/H_2_O = 1:4:15 v/v/v) for 5 min and rinsed with DI water and acetone several times.

### Gold colloids

Gold colloids were prepared using the Frens method. 100 mL of 0.01% (w/w) aqueous HAuCl_4_ solution were heated to boiling point and 0.6 mL of 1% (w/w) of sodium citrate solution were added. In ca. 2 min the boiling solution turned blue (nucleation) and after approximately 5 min the color suddenly changed into red, indicating the formation of spherical gold nanoparticles. After cooling down to room temperature the reaction mixture was centrifuged and 3 mL of concentrated colloidal gold solution were collected from the bottom of the tube.

### Silver colloids

90 mL of aqueous solution of silver nitrate (1.1 mM) were stirred at room temperature. 10 mL of solution containing hydroxylamine hydrochloride (25 mM) and sodium hydroxide (0.1 w/w %) were added. The reaction was completed within a few seconds, which was indicated by a change of solution color to milky yellow. In order to stabilize the silver colloids, 5 mL of aqueous sodium citrate solution (1 w/w %) were added to the final mixture. The reaction mixture was then centrifuged and 3 mL of concentrated colloidal silver solution were collected from the bottom of the tube.

### Metal@TiO_2_ core–shell nanostructures

Metal NPs–titania CSNs were prepared by hydrolysis and polycondensation reaction of titanium(IV) butoxide (TBT). 40 mL of a mixture of ethanol and acetonitrile (50/50 v/v %) were stirred at room temperature. 200 µL of the metal nanoparticle concentrated solution (≈199 µL of water) and 45 µL of the methylamine solution (24.2 µL of water) were added to this mixture. Next, 8 mL of the TBT solution in ethanol were added dropwise (concentrations of TBT in final mixtures are given in Supporting Information File 1, Table S1). In about 15 min the stirred solution turned milky red or milky yellow for gold or silver nanoparticles, respectively. The stirring was kept up for 12 h. After this time, the synthesized CSNs were centrifuged and washed several times with ethanol.

### Tunable resistive pulse sensing (TRPS) measurements

Analogous to the description in [64], TRPS size distribution measurements were carried out using a qNano instrument (Izon Science) with tunable nanopore membranes NP100 (50–200 nm particles size range) for metal colloid measurements and NP150 (100–400 nm particle size range) for the CSNs measurements. The upper and lower cell chambers were filled with an electrolyte (PBS buffer). The arms of the cruciform mount were initially mechanically stretched in the X–Y axis to ≈47 mm and later the X–Y deformation was adjusted for resolution optimization. For all measurements, 40 μL of a suspension of measured particles in the PBS buffer was added to the upper fluid cell compartment, while the lower cell contained pure PBS buffer solution. Experimental conditions, including degree of membrane stretch, applied voltage and pressure, were tuned to optimize the resolution for measurement of each sample. The measurements were conducted for at least 500 particles for each sample. The calibration measurement was carried out after measurement of each sample (with the same conditions) using carboxylated polystyrene nanoparticles (100 nm or 200 nm) supplied by the manufacturer. The statistical data for the particle size distribution, including mean particle diameter, mode particle diameter, max and min particle diameter (*D*_P_10, *D*_P_50 and *D*_P_90) were calculated using the software provided with the instrument.

### UV–vis measurements

The UV–vis extinction spectra were measured at room temperature using a Lambda 900 UV–vis–NIR spectrophotometer (Perkin Elmer) in the 250–800 nm spectral range. Suspensions of the synthesized nanostructures were measured in a 1 cm optical path quartz cuvette placed inside the integration sphere.

### SEM measurements

The morphology of Ag@TiO_2_ and Au@TiO_2_ structures was characterized based on the images obtained using a Quanta 3D FEG dual beam scanning electron microscope. The samples for SEM were prepared by drop-casting suspensions of the core–shell nanostructures on a silicon wafer and drying in air.

## Supporting Information

File 1 Additional experimental data.
TRPS size histograms, images, UV–vis spectra and TBT concentration information.
