# Supplementary material for: Synthesis and characterization of noble metal–titania core–shell nanostructures with tunable shell thickness
Source: Beilstein J Nanotechnol. 2017 Oct 5;8:2083–93. doi: 10.3762/bjnano.8.208 (PMC5647695; doi:10.3762/bjnano.8.208)
Supplement: File 1 — Additional experimental data. TRPS size histograms, images, UV–vis spectra and TBT concentration information. [file Beilstein_J_Nanotechnol-08-2083-s001.pdf]

Supporting Information

for

Synthesis and characterization of noble metal–titania core–shell nanostructures with a tunable shell thickness

Bartosz Bartosewicz[1], Marta Michalska-Domańska[1], Malwina Liszewska[1], Dariusz Zasada[2] and Bartłomiej J. Jankiewicz*[1]

Address: [1]Institute of Optoelectronics, Military University of Technology, Kaliskiego 2 Str. 00-908 Warsaw, Poland and [2]Faculty of Advanced Technologies and Chemistry, Military University of Technology, Kaliskiego 2 Str. 00-908 Warsaw, Poland

Email: Bartłomiej J. Jankiewicz - bartlomiej.jankiewicz@wat.edu.pl

* Corresponding author

Additional experimental data

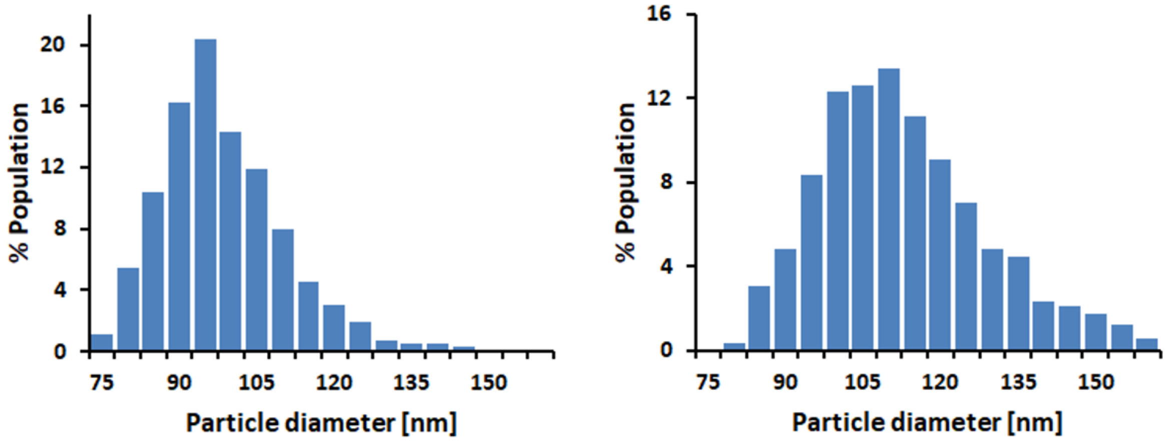

Figure S1: TRPS size histograms of gold (left) and silver (right) nanoparticles.

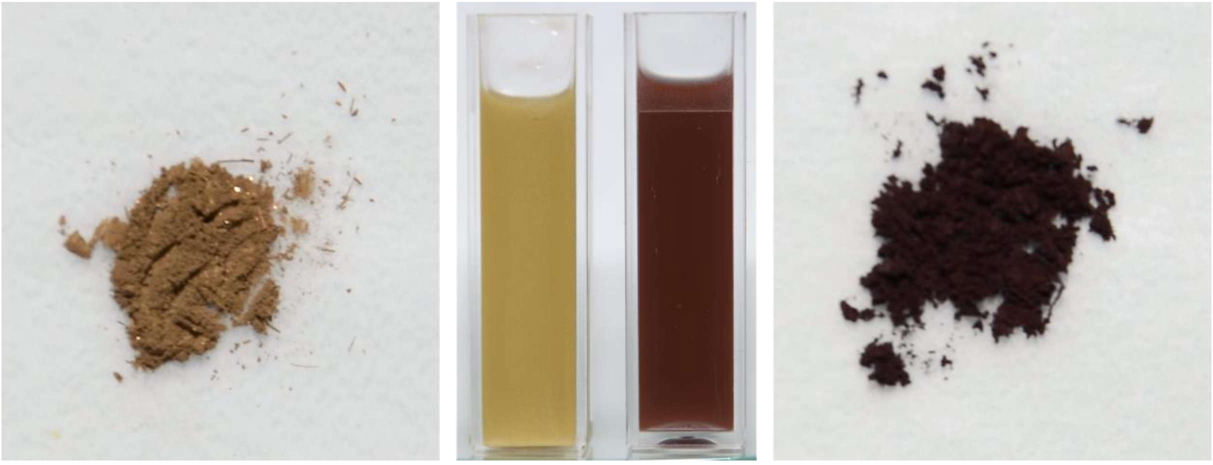

Figure S2: Images of Ag@TiO_2 (left) and Au@TiO_2 (right) core-shell nanostructures in form of powders and water suspensions.

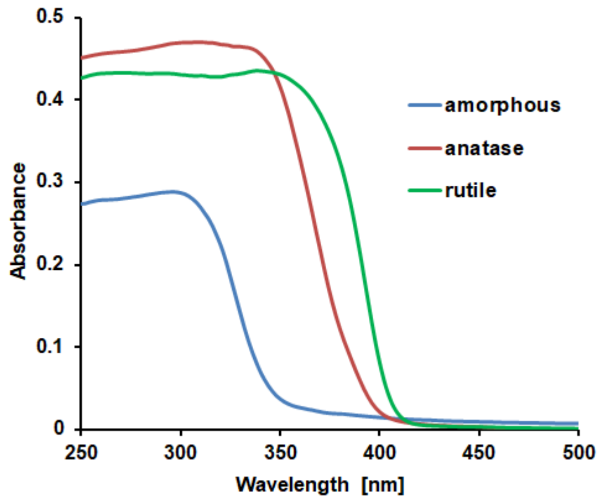

Figure S3: UV–vis spectra of TiO_2 particles in the amorphous and crystalline forms.

Table S1: Concentration of TBT in final mixtures.

**Sample**	A, E	B, F	C, G	D, H
**TBT concentration**	2.0 mM	2.9 mM	3.7 mM	4.6 mM
